# Effect of the Independent Acid Base Variables on Anion Gap Variation in Cardiac Surgical Patients: A Stewart-Figge Approach

**DOI:** 10.1155/2014/907521

**Published:** 2014-02-03

**Authors:** Michalis Agrafiotis, Ilias Keklikoglou, Sofia Papoti, George Diminikos, Konstantinos Diplaris, Vassileios Michaelidis

**Affiliations:** ^1^2nd Department of Intensive Care Medicine, “G. Papanikolaou” General Hospital of Thessaloniki, G. Papanikolaou Avenue, Exohi, 57010 Thessaloniki, Greece; ^2^Department of Cardiothoracic Surgery, “G. Papanikolaou” General Hospital of Thessaloniki, G. Papanikolaou Avenue, Exohi, 57010 Thessaloniki, Greece

## Abstract

*Purpose*. To determine the effect of each of independent acid base variables on the anion gap (AG) value in cardiac surgical patients. *Methods*. This retrospective study involved 128 cardiac surgical patients admitted for postoperative care. The variation of AG (AG_var_) between the day of admission and the first postoperative day was correlated via a multiple linear regression model with the respective variations of the independent acid base variables, that is, apparent strong ion difference (SID_a_), strong ion gap (SIG), carbon dioxide (PCO_2_), and albumin and phosphate concentrations. *Results*. The variations of all the above variables contributed significantly to the prediction of AG_var_ (adjusted *R* ^2^ = 0.9999, *F* = 201890.24, and *P* < 0.001). According to the standardized coefficients (**β**),  SIG_var_ (**β** = 0.948, *P* < 0.001), [Albumin]_var_ (**β** = 0.260, *P* < 0.001), and [Phosphate]_var_ (**β** = 0.191, *P* < 0.001) were the major determinants of AG_var_ with lesser contributions from SID_a, var_ (**β** = 0.071, *P* < 0.001) and PCO_2, var_ (**β** = −0.067, *P* < 0.001). *Conclusions*. All the independent acid base variables contribute to the prediction of the AG value. However, albumin and phosphate and SIG variations seem to be the most important predictors, while AG appears to be rather stable with changes in PCO_2_ and SID_a_.

## 1. Introduction

The anion gap (AG) is a time-honored acid base variable which has been employed for over 30 years as a scanning tool for the presence of unmeasured ions [1]. It is usually estimated as the difference between the commonly measured cations ([Na^+^]+[K^(+)^]) and anions ([Cl^−^]+[HCO_3_^−^]) according to the equation:

(1) $$\begin{array}{c} \text{AG}=\left[{\text{Na}}^{+}\right]+\left[{\text{K}}^{+}\right]-\left[{\text{Cl}}^{-}\right]-\left[\text{HC}{{\text{O}}_{3}}^{-}\right] \end{array}$$

The concept of AG stems from the fundamental principle of electrical neutrality. Hence,

(2) AG=[Na+]+[K+]−[Cl−]−[HCO3−]=[UA−]−[UC+]+[A−]

where [A^−^] denotes the negative charges contributed by the nonvolatile weak acids, mainly albumin and phosphate; [UC^+^] and [UA^−^] represent unmeasured cations and anions, respectively. Other ions participating in the equation as [H^+^], [OH^−^], and [CO_3_^−^] are quantitatively less important and are ignored [1, 2].

The difference of [UA^−^]−[UC^+^] quantifies the total charge contributed by the unmeasured ions and incorporates the concentrations of Ca^2+^, Mg^2+^, lactate, and sulfate which, under normal conditions, are thought to offset each other. An alternative collective term for unmeasured ions is that of strong ion gap (SIG). Thus, in the absence of significant increases in the concentrations of the unmeasured ions, the AG is formed mainly by the negative charges contributed by the nonvolatile weak acids, mostly albumin and to a lesser extend phosphate [1, 2].

A major limitation in the use of AG for the diagnosis and the evaluation of metabolic acidosis is its dependence on the concentrations of the nonvolatile weak acids, of which albumin is the most important [1, 2]. This shortcoming has long been recognized and addressed by various adjustments or corrections, mainly accounting for the deviation of albumin concentration from its reference value [3–5]. An additional issue of concern is the fact that the charges contributed by weak nonvolatile acids may vary with perturbations in the acid base equilibrium. When acidemia ensues, proteins will titrate the excess protons and therefore their net ionic equivalency will be reduced [6]. Indeed, according to the studies by Figge et al. [7–9], over the physiological pH range, the charges contributed by albumin ([Albumin^z−^]) and phosphate ([Phosphate^y−^]) are linear functions of pH:

(3) $$\begin{array}{c} \left[{\text{Albumin}}^{\text{z}-}\right]=\left[\text{Albumin}\right]\cdot \left(0.1204\cdot \text{pH}-0.625\right), \end{array}$$

(4) $$\begin{array}{c} \left[{\text{Phosphate}}^{\text{y}-}\right]=\left[\text{Phosphate}\right]\cdot \left(0.309\cdot \text{pH}-0.469\right), \end{array}$$

where [Albumin] (in g/L) and [Phosphate] (in mmol/L) denote serum albumin and phosphate concentrations, respectively, and charge concentrations are expressed in meq/L.

Therefore, (2) can be recast as

(5) [AG]=[UA−]−[UC+] +[Albumin]·(0.1204·pH−0.625) +[Phosphate]·(0.309·pH−0.469).

The right side of (5) incorporates three variables: the concentration of unmeasured ions (SIG), the concentrations of the non-volatile weak acids ([Albumin] and [Phosphate], collectively termed A_tot_), and pH. However, according to the premises of modern quantitative acid base physiology, pH itself is not an independent variable because it is determined by apparent strong ion difference (SID_a_), SIG, A_tot_, and PCO_2_ [7–11]. Therefore, both the nonrespiratory (SID_a_, SIG, and A_tot_) and as well the respiratory (PCO_2_) component of acid base equilibrium should participate independently in the determination of the AG value. Previous approaches to model AG variation emphasized on the role of the nonvolatile weak acids and they were also based on assumptions or approximations regarding their respective charge concentrations [3–5, 12, 13]. On the other hand, a significant variation of the AG with PCO_2_ was observed in an *ex vivo* experiment on whole blood by Morgan et al. [14].

Given the previous considerations, we embarked on this study to assess the impact and the quantitative significance of each of the independent acid base variables on AG variability *in vivo*.

## 2. Patients and Methods

### 2.1. Study Design

This retrospective study evaluated routine acid base and biochemical data from cardiac surgical patients admitted to our ICU for postoperative care between January 2010 and March 2011. The local Ethics and Scientific Committee approved the study protocol and waived the requirement for informed consent (Ethics and Scientific Committee session number 5/14.03.2012, Chairperson Dr. Ioannis Zarifis). After reviewing the patients' written medical records and the nurses' flowcharts, the following paired acid base and biochemical data from the day of admission and the first postoperative day were extracted: pH, PCO_2_, Na, K, and Cl (from the output of the blood gas machine) and serum albumin and phosphate concentrations (from the biochemical report). A time interval of 16–18 hours intervened between consecutive measurements. In addition, we recorded clinical and demographic data from each patient, including age, sex, type of operation, and logistic Euroscore value.

Blood gas sampling was performed with the use of specifically designed commercially available syringes which come prefilled with dry electrolyte-balanced heparin (PICO sampler; Radiometer, Copenhagen, Denmark); the first 2-3 mL of blood was discarded to avoid contamination with the flushing fluid. Biochemical samples were obtained from the radial artery catheter immediately after the first blood gas samples were drawn. The blood gas samples were analyzed at 37°C for blood gases and electrolytes in the point-of-care blood gas and electrolyte analyzer (ABL800 FLEX analyzer; Radiometer, Copenhagen, Denmark). Albumin and phosphate concentrations were assessed in the hospital central laboratory using colorimetric techniques (Olympus EU 640; Olympus, Center Valley, Pennsylvania, USA).

Following the methodology employed by Park et al. [15], we developed a multiple linear regression model correlating AG variation between admission and first postoperative day with the respective variations of SID_a_, SIG, PCO_2_, [Albumin], and [Phosphate]. The variation of any acid base variable (e.g., *X*) between the day of admission and the first postoperative day was defined according to the following formula:

(6) $$\begin{array}{c} X_{\text{var}}=X_{1}-X_{0}, \end{array}$$

where the suffix var denotes variation and the suffixes 0 and 1 correspond to the day of the admission and the first postoperative day, respectively.

### 2.2. Mathematical Acid Base Calculations

Quantitative acid base analysis was based on the principles advanced by Stewart [10, 11] and subsequently modified by Figge et al. [7–9] to model the effects of proteins and other nonvolatile weak acids on acid base equilibria. For each patient, the following acid base variables were assessed: AG, bicarbonate concentrations, albumin and phosphate charge concentration, apparent and effective strong ion difference, and strong ion gap (SIG). AG (in meq/L) was calculated by (1), while bicarbonate concentration (in mmol/L) was calculated by the Henderson-Hasselbalch equation:

(7) $$\begin{array}{c} \left[\text{HC}{{\text{O}}_{3}}^{-}\right]=0.0301\cdot {\text{PCO}}_{2}\cdot 10^{\left(\text{pH}-6.1\right)}, \end{array}$$

where PCO_2_ is expressed in mmHg.

Albumin and phosphate charge concentrations were calculated according to (3) and (4), respectively. Since, in the AG formalism Ca^2+^, Mg^2+^ and lactate are considered unmeasured ions and therefore incorporated into SIG calculation, the equation for SID_a_ (in meq/L) was cast in a more simplified form:

(8) $$\begin{array}{c} {\text{SID}}_{\text{a}}=\left[{\text{Na}}^{+}\right]+\left[{\text{K}}^{+}\right]-\left[{\text{Cl}}^{-}\right]. \end{array}$$

Last, effective strong ion difference (SID_e_) and SIG (all in meq/L) were calculated according to the following equations:

(9) $$\begin{array}{c} {\text{SID}}_{\text{e}}=\left[{\text{Albumin}}^{\text{z}-}\right]+\left[{\text{Phosphate}}^{\text{y}-}\right]+\left[\text{HC}{{\text{O}}_{3}}^{-}\;\right], \end{array}$$

(10) $$\begin{array}{c} \text{SIG}={\text{SID}}_{\text{a}}-{\text{SID}}_{\text{e}}. \end{array}$$

### 2.3. Statistical Analysis

Statistical analysis was performed using the Statistical Package for the Social Sciences software (SPSS Inc., Chicago, Illinois, release 17.0). Continuous variables are expressed as mean ± standard deviation (SD), unless stated otherwise, and dichotomous (categorical) variables are expressed as frequency counts (proportions).

The normality of data distribution was assessed by inspection of histograms. The multiple linear regression model was built in the forward mode with entry and removal criteria of *P* = 0.05 and *P* = 0.10, respectively. Pearson's statistic was employed to assess the presence of single collinearity between the independent variables, with an *R* value > 0.85 chosen as a single collinearity criterion. Multicollinearity was suggested by a tolerance value less <0.1 and/or a variance inflation factor (VIF) > 10. We plotted the residuals against the dependent and the independent variables to disclose any possible nonlinear relationship. The change in *F* statistic was used to assess the significance of *R* ^2^ improvement by the inclusion of a new variable in the model and a *P* value less than 0.05 was considered to be significant.

## 3. Results

One hundred and twenty eight cardiac surgical patients (age 65.8 ± 10 years, 103 males) were included in study. Clinical and demographic characteristics of the patients are summarized in Table 1. The values of AG and of its predictors and their respective variations between admission and the first postoperative day are presented in Table 2.

The output of the multiple linear regression model is summarized in Table 3. According to the standardized correlation coefficients (*β*) of the model, AG_var_ can be independently predicted by SIG_var_ (*β* = 0.948, *P* < 0.001), [Albumin]_var_ (*β* = 0.260, *P* < 0.001), [Phosphate]_var_ (*β* = 0.191, *P* < 0.001), SID_a,var_ (*β* = 0.071, *P* < 0.001), and PCO_2,var_ (*β* = −0.067, *P* < 0.001). This combination of independent variables virtually explained the whole variance of the dependent variable with an adjusted *R* ^2^ = 0.9999 (*F* = 201890.24, *P* < 0.001).

## 4. Discussion

In this study we endeavored to identify the independent predictors of AG variation and assess their quantitative importance. The results of our multiple linear regression model suggest that AG variation is determined mainly by two factors: the concentration of the unmeasured ions and the concentrations of nonvolatile weak acids, namely, albumin and phosphate. An important finding of our study is that strong ion difference and PCO_2_ also participate independently in the prediction of AG variation although their contributions are quantitatively less important. Indeed, it should be noted that, within the usual clinical settings, the variations of SID_a_ and PCO_2_ are unlikely to be the cause of significant bias in the AG value. For instance, assuming a zero change for the other parameters, a decrease in PCO_2_ by 10 mmHg or an increase in SID_a_ by 10 meq/L would increase AG value by 0.42 and 0.65 meq/L, respectively. Interestingly, in an* ex vivo* experiment on whole blood by Morgan et al. [14], in which PCO_2_ was varied from >200 to <20 mmHg, the calculated average increase in AG per mmHg was 0.03 and 0.02 meq/L for normal and diluted blood, respectively (calculations based on mean initial and final values). These values are of the same order with the correlation coefficient for PCO_2,var_ in our model (Table 3).

To our knowledge, this is the first study that evaluated the role of respiratory perturbations on the value of the AG *in vivo*. The results of this study indicate that respiratory acid base disorders do not impact a quantitatively significant bias on the AG value. Therefore, the AG (particularly in its albumin and phosphate-adjusted form) can be reliably used for the assessment of metabolic acid base disorders in patients with respiratory acid base disturbances. Of note, an alternative metabolic acid base index, standard base excess, was recently demonstrated to exhibit a quantitatively significant variability with changes in PCO_2_ [15].

On the other hand, the possibility that changes in SID_a_ may influence the AG value has not been considered so far in quantitative acid base analyses. This fact is likely to be partly related to the persistence of previous firmly established premises of traditional acid base physiology. Thus, the traditional classification of acidosis distinguished between the “hyperchloremic” and the “nonhyperchloremic” types and ignored the other strong ions, while the change in [Cl^−^] was thought to be more or less cancelled out by an opposite change in [HCO_3_^−^], essentially leaving AG unaltered (non-AG acidosis) [16].

Furthermore, although the homeostases of Cl^−^ and CO_2_ are thought to be interlinked at the level of the erythrocytes [17] and the kidneys [18], we have found that the variations of SID_a_ and PCO_2_ between admission and the first postoperative day predict independently AG variation without significant multicollinearity. This is not surprising since, according to Stewart-Figge theory [7–11], within a single compartment, both PCO_2_ and SID_a_ should be independent predictors of acid base equilibrium, regardless how their values were established. In addition, the opposite correlation coefficients of PCO_2_ and SID_a_ (Table 3) preclude the possibility of renal or tissue compensation.

Based on the output of the model (Table 3), the change of AG value from a reference state can be partitioned into the respective changes of the four independent acid base variables according to the following equation:

(11) ΔAG=0.934·ΔSIG  +0.251·Δ[Albumin] +1.704·Δ[Phosphate]+0.065·ΔSIDa   −0.042·ΔPCO2,

where Δ denotes change or difference.

If the quantitatively minimal contributions from SID_a_ and PCO_2_ are ignored and the correlation coefficients are rounded, our model's master equation (11) can be recast in a more simplified form:

(12) ΔAG=ΔSIG+0.25·Δ[Albumin] +1.7·Δ[Phosphate].

Alternatively we can write

(13) ΔAG−ΔSIG=0.25·Δ[Albumin] +1.7·Δ[Phosphate].

Equation (13) quantifies the partition in the change of AG value that is not attributed to the addition of unmeasured ions. Therefore, the AG value can be adjusted to reference conditions according to the following equation:

(14) AGadj=AGob+0.25·([Albumin]ref−[Albumin]ob) +1.7·([Phosphate]ref−[Phosphate]ob),

where the suffixes ref, adj, and ob denote the reference, adjusted, and observed values, respectively. With respect to albumin, this adjustment is identical to the one proposed by Figge et al. [3], although in their study on critically ill patients the contribution of phosphate was not modeled. Alternatively, Carvounis and Feinfeld [5] suggested that the adjustment factor should be expressed either as 1.5 · ([Albumin]_ref_ − [Albumin]_ob_) for patients with total CO_2_ > 21 meq/L or as 2 · ([Albumin]_ref_ − [Albumin]_ob_) for patients with total CO_2_ < 22 meq/L. More recently, the slope of AG versus albumin was found to be equal to 0.23 meq/L per g/L of albumin in study by Feldman et al. [12] involving a large database of in- and outpatients.

If we integrate both sides of (12) and solve for SIG, we can also obtain the unmeasured ion concentration:

(15) SIG=AGob−(0.25[Albumin]ob      +1.7[Phosphate]ob).

It should be noted here that Kellum proposed a similar empirical calculation rule for the unmeasured ion concentration (termed corrected AG), by averaging albumin and phosphate charges over the acidemic pH range and subtracting them from the observed AG value [4]. Hence,

(16) AGcor=AGob−(0.2[Albumin]ob     +1.5[Phosphate]ob),

where the suffix cor denotes the corrected AG value. Again, the units for albumin and phosphate concentrations are g/L and mmol/L, respectively.

The validity of Figge's algorithm for the correction (adjustment) of AG value according to albumin concentration has been assessed in several clinical studies. Generally, studies in critically ill [19–21] and in hospitalized patients [22] have demonstrated that albumin-corrected AG (ACAG) is not specific enough for the detection of hyperlactemia although it may rule out its presence [19]. In addition, in a study by Hatherill et al. on critically ill children with shock, the difference between the observed ACAG and its reference value could predict an increase >5 mmol/L in occult tissue ions (lactate plus unmeasured ions) with a sensitivity of 87% and a specificity of 75% [23]. On the other hand, a study by Moviat et al. [21] noted that when the difference between the observed ACAG and its reference value was adjusted for lactate, it performed reasonably well in the prediction of SIG in critically ill adult patients (bias 1.86 and precision 0.96 according to Bland-Altman analysis).

Our approach is limited by the fact that we did not prospectively validate our model on an independent patient population. However the formalisms derived from this model (see (14) and (15)) do not differ significantly from previous approximations employed for the adjustment or the correction of the AG value [3, 4].

In addition, we should point out that this mathematical model is only applicable within the ranges of the independent variable variations observed in this study. On the other hand, a major advantage of our approach is that it does neither require nor resort to assumptions or approximations regarding the pH value or albumin and phosphate charge concentrations. Only the knowledge of the independent variables of acid base equilibrium, that is, SID_a_, [Albumin], [Phosphate], SIG, and PCO_2_, suffices for the prediction of AG value.

To conclude, we have developed a comprehensive mathematical model which correlates AG variation with the respective variations of SIG, [Albumin], [Phosphate], SID_a_, and PCO_2_. All the above acid base variables exert an independent influence on the AG value, although SIG, [Albumin], and [Phosphate] are quantitatively the most important predictors. Moreover, the AG seems to be a robust index for the assessment of metabolic acid base disorders in patients with coexistent respiratory or strong ion acid base disturbances.

## Figures and Tables

**Table 1:** Clinical and demographic characteristics of patients (*N* = 128).

Age (years)	65.8 ± 10.0
Males, *n*/*N* (%)	103/128 (80.4)
Logistic Euroscore	4.65 (0.88, 78.52)*
Types of operations, *n*/*N* (%)	
CABG	90/128 (70.3)
Valvular	16/128 (12.5)
Aortic	8/128 (6.2)
Combined CABG and valvular	7/128 (5.5)
Other	7/128 (5.5)

*Median (range).

CABG: coronary artery bypass grafting.

**Table 2:** Variation of anion gap and its predictors between admission and first postoperative day (*N* = 128).

Acid base variables	Admission	First postoperative day	Variation
SID_a_, meq/L	37.8 ± 4.0	37.4 ± 3.8	−0.4 ± 4.9
PCO_2_, mmHg	40.1 ± 5.8	37.7 ± 4.6	−2.4 ± 7.0
Phosphate, mmol/L	1.1 ± 0.4	1.2 ± 0.4	0.05 ± 0.5
Albumin, g/L	31.5 ± 5.0	29.5 ± 4.7	−1.9 ± 4.7
SIG, meq/L	4.0 ± 4.7	2.5 ± 3.8	−1.5 ± 4.5
AG, meq/L	14.3 ± 4.5	12.6 ± 3.5	−1.7 ± 4.5

**Table 3:** Output of the multiple linear regression model correlating AG_var_ (dependent variable) with SIG_var_, [Albumin]_var_, [Phosphate]_var_, PCO_2,var_, and SID_a,var_ (independent variables) (adjusted *R* ^2^ = 0.9999, *F* = 201890.24, and *P* < 0.001).

	Unstandardized coefficients (95% CI)	Standardized coefficients	*P* value	Tolerance	VIF
Constant	0 (−0.014, 0.012)		0.883		
SIG_var_, meq/L	0.934 (0.931, 0.938)	0.948	<0.001	0.310	3.228
[Albumin]_var_, g/L	0.251 (0.248, 0.253)	0.260	<0.001	0.680	0.1471
[Phosphate]_var_, mmol/L	1.704 (1.685, 1.723)	0.191	<0.001	0.821	1.218
PCO_2,var_, mmHg	−0.042 (−0.044, −0.041)	−0.067	<0.001	0.623	1.606
SID_a,var_, meq/L	0.065 (0.062, 0.068)	0.071	<0.001	0.283	3.530

CI: confidence intervals.
